# The Promoter SNPs Were Associated with Both the Contents of Poly-Unsaturated Fatty Acids (PUFAs) and the Expressions of PUFA-Related Genes in Common Carp

**DOI:** 10.3390/biology12040524

**Published:** 2023-03-30

**Authors:** Yan Zhang, Zi-Ming Xu, Qi Wang, Qing-Song Li, Xiao-Qing Sun, Jiong-Tang Li

**Affiliations:** 1 National Demonstration Center for Experimental Fisheries Science Education, Shanghai Ocean University, Shanghai 201306, China; 2 Key Laboratory of Aquatic Genomics, Ministry of Agriculture and Rural Affairs, Beijing Key Laboratory of Fishery Biotechnology, Chinese Academy of Fishery Sciences, Beijing 100141, China

**Keywords:** association study, common carp, fatty acid elongase, promoter, poly-unsaturated fatty acid

## Abstract

**Simple Summary:**

Fish is an important PUFA source for human diet. Much research has focused on increasing the PUFA products of fish. Improving the biosynthesis of PUFAs would also be a way to contribute to nutrition supplement for human health. Aquaculture selective breeding by genetic markers is essential for increasing the content of PUFAs. Fatty acid desaturase 2 (*fads2*) and elongase 5 (*elovl5*) have been proven key rate-limiting enzymes in the synthesis of PUFAs for bony fishes. These genes’ coding SNPs (cSNPs) were reported to be significantly associated with the PUFA contents in common carp. However, the promoter SNPs (pSNPs) effectiveness for the PUFA contents has not been evaluated yet. This study identified the genetic variants in the promoter regions of *fads2a/2b* and *elovl5a/5b*. Moreover, the joint effects of multiple cSNPs and pSNPs in *fads2b* and *elovl5b*, two major genes related to the PUFA biosynthesis, were evidenced by the higher explained percentages of phenotype. Furthermore, the gene expression level of *fads2a* and *fads2b* showed significant positive correlation with the contents of multiple PUFAs. These SNPs would be potential markers for future selection to improve the PUFA contents in common carp.

**Abstract:**

The allo-tetraploid common carp encodes two duplicated *fads2* genes (*fads2a* and *fads2b*) and two duplicated elovl5 genes (*elovl5a* and *elovl5b*). The coding SNPs (cSNPs) of these genes were reported to be significantly associated with the PUFA contents. Whether the promoter SNPs (pSNPs) were associated with the PUFA contents has not been reported yet. In this study, after sequencing the promoters of these four genes, we identified six pSNPs associated with the contents of PUFAs in common carp, including one *elovl5a* pSNP, one *elovl5b* pSNP, and four *fads2b* pSNPs. The pSNPs were predicted in the locations of transcriptional factor binding sites. Together with previously identified cSNPs in *fads2b* and *elovl5b*, the pSNPs and cSNPs of these two genes had the joint effects on the PUFA contents with higher explained percentage of phenotypic variation of the PUFA contents than single gene. The expression levels of both *fads2a* and *fads2b* were significantly positively correlated with the contents of six PUFAs. The *fads2b* pSNPs corresponding to higher *fads2b* expression levels were associated with higher PUFA contents. The pSNPs and cSNPs will be useful for the future selection breeding of common carp with higher PUFA contents.

## 1. Introduction

The poly-unsaturated fatty acids (PUFAs) are involved in numerous critical biological processes [1,2,3]. Fish is an important PUFA source for human diet and is essential for human health [4,5]. The PUFAs in fish were acquired from both dietary and biosynthesis. The levels of fish oil in the feed were important for the PUFA content in fish [6,7]. However, the limited availability of fish oil becomes a critical bottleneck in aquaculture feed production systems. Selective breeding of fish effectively biosynthesizing endogenous PUFAs would reduce the fish oil requirement and decrease the breeding cost.

Some freshwater fish have the biosynthesis ability of PUFAs by converting C18 PUFAs to longer-chain PUFAs [8,9]. Fatty acid desaturase (Fads) and elongases of very long chain fatty acids (Elovl) are two functional enzyme families in the PUFA biosynthesis pathway. They participate in the PUFA biosynthesis by desaturation and elongation, respectively [10,11]. In fish, *fads2* was responsible for introducing double bonds at specific positions of the fatty acyl chain [12]. The elovl gene family in fish includes multiple elovl genes such as *elovl2*, *elovl4*, and *elovl5*, which have specific substrates in the elongation of PUFAs [13]. The SNPs in the coding and promoter regions of *fads2* and *elovl5* have been proven to be associated with the PUFAs content in human [14], beef cattle [15], bovine milk [16,17], pigs [18], and gilthead sea bream [19]. Therefore, investigating the SNPs in the coding and promoter regions of *fads2* and *elovl5* would benefit for the selective breeding of fish with high PUFA contents.

Common carp (*Cyprinus carpio*) is cultured worldwide [20]. Common carp lipids are composed of 21.5% PUFA, including 1.3–6.7% n-3 FA and 1.3–14.8% n-6 FA [21,22], suggesting there is much variance of PUFA content among carp strains. Different from the diploid freshwater fish, common carp is an allotetraploid cyprinid species and encodes two duplicate *fads2* genes (*fads2a* and *fads2b*) and two duplicated *elovl5* genes (*elovl5a* and *elovl5a*) [23]. We previously identified the coding SNPs (cSNPs) of these four genes associated with the PUFAs content in common carp [24,25]. However, the promoter SNPs (pSNPs) of these genes have not been studied yet. Furthermore, whether there exists the joint effect of cSNPs and pSNPs of these four genes on the PUFAs contents was also still unknown.

To better understand these questions, we sequenced the promoter regions of common carp *fads2a*, *fads2b*, *elovl5a*, and *elovl5b* and detected the pSNPs of these genes, and then expected to identify the pSNPs significantly associated with the PUFAs content. Further, we also studied the joint effects the cSNPs and pSNPs of these genes on the PUFA contents. The pSNPs and cSNPs would be used as biomarkers for the selective breeding of common carp with high PUFAs content.

## 2. Materials and Methods

### 2.1. Sampling and Quantifying PUFA Content

Previously we measured the PUFA contents of 269 one-year-old common carp from three strains, including 124 individuals of the Furui strain (FRC), 98 individuals of the Jian strain (JC), and 47 individuals of the Huanghe strain (HHC) [24]. Briefly, the freeze-dried muscle powders were dissolved in NaOH-methanal and then the boron trifluoride-methanol solution. The fatty acid methyl esters (FAMEs) were dried with the oxygen-free nitrogen and then resuspended with hexane. The solution was analyzed by gas chromatography on an Agilent 7890A system (Agilent Technologies, Santa Clara, CA, USA).

### 2.2. Amplifying and Sequencing the Promoters of Common Carp fads2a, fads2b, elovl5a, and elovl5b

The genomic DNA of each sample was extracted from the muscle with TIANamp Genomic DNA Kit (TIANGEN Biotech, Beijing, China). The concentration and quality of DNA were determined with NanoDrop 2000 spectrophotometer (Thermo Scientific^TM^, Waltham, MA, USA) and 1% agarose gel electrophoresis. The full-length transcripts of common carp *fads2a*, *fads2b*, *elovl5a*, and *elovl5b* were aligned to the recent chromosome level of common carp (GenBank accession number, NC_056596.1:2766266–2766294, NC_056621.1:4466320–4465379, NC_056584.1:836157–835458, and NC_056609.1:5220304–5221380). The promoter region of each gene was deemed as the 500 bp upstream region of the first exon [26]. The PCR primers were designed using Primer Premier software [27] to specifically amplifying the promoters (Supplementary Table S1). The PCR was performed with 2 × EasyTaq SuperMix (Tiangen, Beijing, China). The PCR reactions included an initial denaturation of 94 °C for 4 min, followed by 35 cycles of 94 °C for 30 s, specific temperature for 30 s and 72 °C for 1 min, and with a final extension step at 72 °C for 10 min (Supplementary Table S1). The PCR products were determined on 1% agarose gel and then purified with EasyPure Quick Gel Extraction Kit (TransGen Biotech, Beijing, China). All the purified PCR products were sequenced with a paired-end Sanger sequencing mode on an ABI 3730 XL machine (TaiheGene, Beijing, China).

### 2.3. Genotyping and Estimating the Genetic Diversities

We aligned the sequences to the genome using NCBI Blastn. The sequence redundancy resulted from whole genome duplication might lead to mis-amplification. Therefore, if one sequence was aligned to its corresponding promoter with the highest score, this sequence was retained in the following analysis. To call the pSNPs, the confirmed sequences were aligned to the corresponding promoters using the novoSNP software [28]. The pSNPs were featured with F-scores ≥ 30 and at least two sequencing peaks of one site per sample. The homozygotes and heterozygotes were auto-detected with this software.

We grouped three strains into one population and calculated the genetic diversities of the pSNPs, which had a frequency over 0.03. For each pSNP, the observed heterozygosity (Ho), and the expected heterozygosity (He) were measured with the Genepop software 4.7 [29]. The polymorphism information content (PIC) was calculated using PICcalc 0.6 [30].

We predicted the potential transcriptional factors (TFs) and their binding sites (TFBSs) in each promoter using TFBIND (https://tfbind.hgc.jp/, accessed on 10 December 2022) with the default parameters [31]. If one pSNP was located in the TFBSs, it was considered as a TFBS-related pSNP.

### 2.4. Association between the pSNPs and the PUFA Contents

To identify the associated pSNPs with the PUFA contents, we used analysis of variance (ANOVA) and general linear model (GLM) analysis with 100,000 permutations in Tassel 5 [32], respectively. All samples were classified into different groups based on their genotypes in one pSNP. With ANOVA, we performed the pairwise comparison between any two groups on each PUFA content.

Each ANOVA *p* value was corrected using the false discovery rate (FDR) method for multiple hypothesis testing. The GLM model was performed with the genetic distance matrix and 100,000 permutations. One pSNP was significantly associated with the content of one PUFA if it had a *p* value < 0.05 in the GLM method and an FDR corrected *p* value < 0.1 in the ANOVA. The explained percentage of phenotypic variation (PV) of each pSNP was measured using Tassel 5.

To study the strain effect on the association study, we performed the GLM analysis incorporated with the population structures from the PCA analysis (GLM-PCA). We also used the analysis of covariance (ANCOVA) with the strain information to estimate the putative strain effect.

### 2.5. Joint Effect of the pSNPs and cSNPs of elovl5b and fads2b on the PUFA Contents

Previously we demonstrated the joint effects of 25 cSNPs from *elovl5b* and *fads2b* on the contents of 12 PUFAs. The *fads2b* and *elovl5b* were two major effect genes on the contents of multiple PUFAs since they had more associated cSNPs with the contents of at least two PUFAs [24,25]. We further studied the joint effect of cSNPs and pSNPs of these two genes on the PUFA contents. Before the analysis, we studied the linkage disequilibrium (LD) between any two compared SNPs in one gene with PLINK [33]. The LD was represented with the D’ value. The SNPs were clustered into one haplotype block if the D’ value of any two compared SNPs was over 0.9. If multiple SNPs were attributed into one haplotype block, the joint effect of multiple SNPs might be overestimated. Therefore, we retained one SNP significantly associated with the PUFA content to represent the SNPs in the same block. The joint effect of multiple retained SNPs was estimated with the explained percentage of PV. We generated three SNP sets, including (i) cSNPs and pSNPs in *fads2b* (F2b.cpSNPs); (ii) cSNPs and pSNPs in *elovl5b* (E5b-cpSNPs); (iii) cSNPs and pSNPs in *fads2b* and *elovl5b* (F2b-E5b-cpSNPs). In each SNP set, we generated different genotype combination. If one genotype combination was observed in at least three individuals, this combination was used in the comparison. For each PUFA, we performed the pairwise comparisons of the PUFA contents among different retained combinations using ANOVA. The explained percentages of PV of the genotype combination to each PUFA content was estimated with the function of ‘lm’ in R [34].

### 2.6. Correlation between the Expression Level of Each Gene and the PUFA Content

The livers of 44 randomly selected individuals were used to extract the total RNA with TRIzol (Invitrogen, Waltham, MA, USA). The liver is the major organ where the PUFAs are biosynthesized [8]. The concentration of RNA was determined using the NanoDrop 2000 spectrophotometer (Thermo Scientific™, Wilmington, DE, USA), and its quality was determined by 1% agarose gel electrophoresis. The RNA was reverse transcribed to cDNA using the SuperScript III RNase H-Reverse Transcriptase Kit (Invitrogen, Waltham, MA, USA) with oligo-dT. The common carp β-actin (GenBank accession: M24113.1) was used as an endogenous control. We designed the gene-specific primers to quantify the expression level of targeted gene (Supplementary Table S2). The expression level of the targeted gene in the liver was measured with real-time quantitative PCR (RT-qPCR). The qPCR was performed in triplicate on an ABI7500 (Thermo Scientific™, Wilmington, DE, USA) using the SYBR Green Realtime PCR Master Mix (TOYOBO, Osaka, Japan). The reaction was performed in 15 µL of reaction volume containing 7.5 µL mix, 1 µL cDNA, 0.3 µL forward and reverse primers, and 5.9 µL nuclease-free water. The qPCR profile contained an initial activation step at 95 °C for 2 min, followed by 40 cycles: 15 s at 95 °C, 15 s at 60 °C and 30 s at 72 °C. The product sizes were checked by agarose gel electrophoresis. The expression of beta-actin was used to normalize the target gene expression level. For each gene across 44 samples, we calculated the Pearson correlation coefficient and the associated *p* value between its expression level and each PUFA content. Finally, we used the FDR method to adjust the *p* values for multiple testing.

For each pSNP, the samples were classified into different groups based on their genotypes. The normalized expression levels of the corresponding gene in these individuals were distributed into different groups.

## 3. Results

### 3.1. The Genetic Diversities of the Promoters of fads2a, fads2b, elovl5a, and elovl5b

The promoter sequences of *fads2a*, *fads2b*, *elovl5a*, and *elovl5b* were successfully sequenced in 228 individuals (Supplementary Table S3). The lengths of amplified sequences ranged from 698 bp to 1077 bp. The A + T content of these four gene promoter sequences was 63.93%, 64.54%, 63.88%, and 57.02%, respectively. The promoters between the two fads2 genes had an identity of 84.19% and the identity between the promoters of elovl5a and elovl5b was 74.49%.

The pSNPs positions were numbered according to their positions relative to the first base of the full-length mRNA sequences of *fads2a*, *fads2b*, *elovl5a*, and *elovl5b* (GenBank accession numbers: MK852165.1, MK852166.1, MK893918.1, and MK893919.2). With the minor allele frequency (MAF) over 0.03 and the allowed missing genotypes per individual less than 25%, nineteen pSNPs were identified in *fads2a*, *fads2b*, *elovl5a*, and *elovl5b* genes in 208 individuals, including one in *fads2a*, eleven in *fads2b*, five in *elovl5a*, and two in *elovl5b* (Table 1).

The observed heterozygosities (Ho) of 19 loci ranged from 0.049 to 0.458 where only five pSNPs had the Ho values smaller than 0.1. The expected heterozygosities (Ho) of these loci were from 0.072 to 0.5 and few pSNPs had the He values smaller than 0.1. Similarly, the PIC value of only one pSNP was smaller than 0.1. The number of pSNPs having the diversity rates (Ho, He, and PIC) higher than 0.1 was more than the ones of cSNPs in these four genes [24,25], suggesting higher polymorphic levels in the promoters than the coding regions. Among 19 pSNPs, two had two genotypes per locus while 17 pSNPs had three genotypes per locus. The phenomena were different from the observation in the cSNPs of these four genes, where few cSNPs had three genotypes per locus. This data also supported the higher polymorphic levels in the promoters.

### 3.2. Associations between the pSNPs with Common Carp PUFA Contents

**Figure 1:**
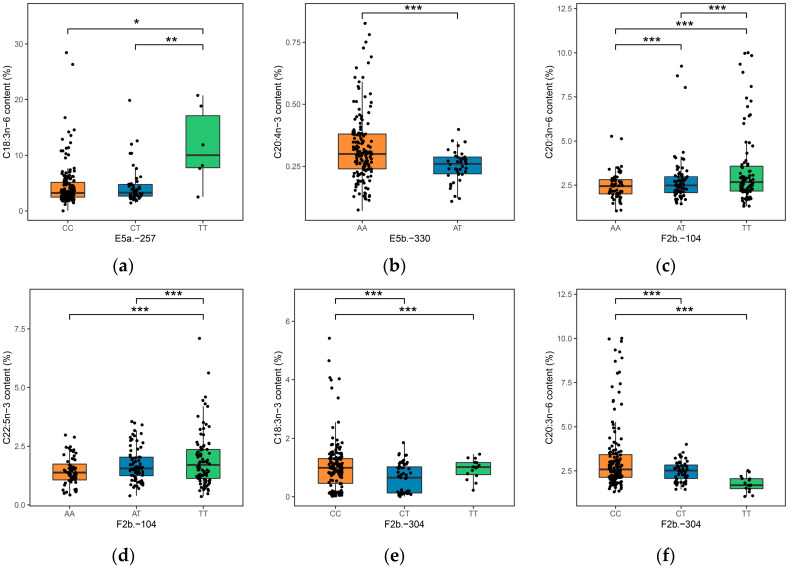

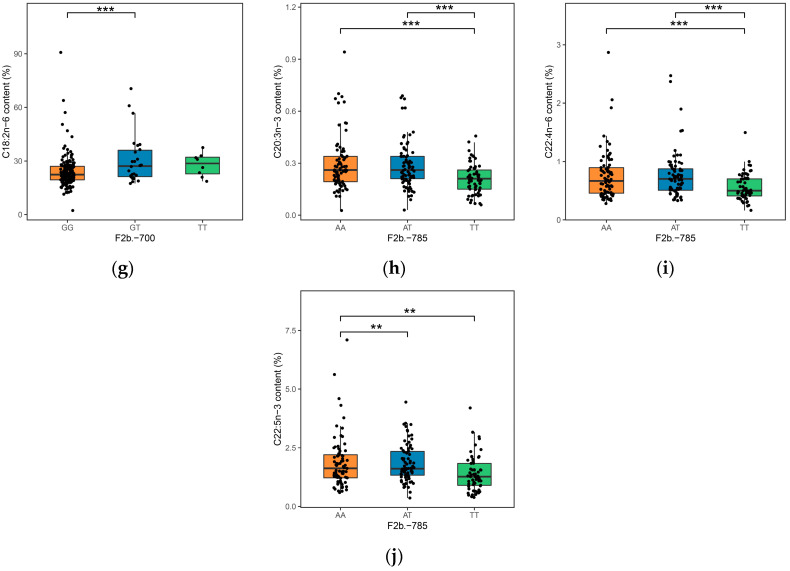
Box plots showing different PUFA content differences among distinct genotypes. (**a**): E5a.-257; (**b**): E5b.-330; (**c**,**d**) F2b.-104; (**e**,**f**) F2b.-304; (**g**) F2b.-700; (**h**–**j**) F2b.-785. * represented FDR *p* values < 0.05. ** meant FDR *p* values < 0.01. *** indicated FDR *p* values < 0.001.

We did not observe the pSNPs in *fads2a* associated with the PUFA contents. For *elovl5a* and *elovl5b*, only one pSNP in each gene was associated with the content of one PUFA (Figure 1a,b). Intriguingly, four *fads2b* pSNPs had an association with the contents of six PUFAs. Three pSNPs, including F2b.-104, F2b.-304, and F2b.-785, were significantly associated with the contents of more than two PUFAs (GLM analysis *p* value < 0.05, Table 2). These six pSNPs were in the transcription factor binding sites (TFBSs) with predicated scores over 0.75 (Supplementary Table S4). The transcription factors (TFs) including HNF, NFKB, Sp1, and C/EBPα were proven to regulate the expressions of *fads2* and *elovl5* [35,36,37].

Combining different strains into one population might introduce the strain effect on the association study. The GLM analysis and ANOVA identified ten significant associations, eight of which were confirmed by the GLM-PCA analysis (*p* values listed in Supplementary Table S5). The ANCOVA analysis with the strain information validated all ten associations (Supplementary Table S6). These two results indicated that the mixed population with more samples was suitable for the association study to increase the accuracy.

F2b.-104 showed associations with the contents of C20:3n-6 and C22:5n-3 (*p* values listed in Figure 1c,d). This locus included three genotypes, two homozygous genotypes (TT and AA) and the heterozygous genotype (AT). Interestingly, in these two associated PUFAs, the individuals with the homozygous genotype (TT, major allele) had higher PUFA contents than the samples of the other groups. The fold changes of mean PUFA contents between the highest content group and the lowest content group were 1.38 (C20:3n-6) and 1.34 (C22:5n-3), respectively.

F2b.-304 was associated with the contents of C18:3n-3 and C20:3n-6 (Figure 1e,f). In this locus, three genotypes including two homozygous genotypes (CC and TT) and the heterozygous genotype (CT) were observed. Likewise, the individuals with the homozygous genotype (CC, major allele) had the highest PUFA contents. The fold changes of mean PUFA contents between the highest content group and the lowest content group were 1.1 (C18:3n-3) and 1.81 (C20:3n-6), respectively.

F2b.-785 was associated with the contents of C20:3n-3, C22:4n-6, and C22:5n-3 (Figure 1h–j). This locus also had three genotypes. The homozygous individuals of the major allele and the heterozygous samples had close contents of these three PUFAs, which were higher than the contents in the homozygous individuals of the minor alleles.

### 3.3. Joint Effect of pSNPs and cSNPs from fads2b and elovl5b on the PUFA Contents

Previously we found that *fads2b* and *elovl5b* might be two major genes responding to common carp PUFA contents [24,25]. Herein, we estimated the joint effects of the pSNPs and cSNPs in these two genes on the contents of multiple PUFAs.

We observed three haplotype blocks in *fads2b* (Supplementary Table S7). Two *fads2b* pSNPs associated with the PUFA contents, including F2b.-700 and F2b.-785, were attributed into one block and we retained F2b.-785 in the joint effect analysis. However, the other pSNPs and cSNPs were not clustered into the same blocks and thus kept in the following analysis. Likewise, all *elovl5b* pSNPs and cSNPs cannot be grouped into the same blocks. These SNPs were used in the joint effect analysis.

The cpSNP set of one gene explained more percentages of PV than single SNP in the same gene. The pSNP of E5b.-330 had an explained PV of 4.53% on C20:4n-3. The PV of the cSNP of E5b.172 was 7.12%. The PV of the cpSNPs of E5b increased to 13.71%. Similar observations of increasing PV values were found in both the contents of the other PUFAs and the cpSNPs of F2b.

The results also showed higher explained PV values of F2b.cpSNPs than E5b.cpSNPs on the contents of eleven PUFAs (Table 3 and Table 4). This data suggested that *fads2b* should be the predominant gene affecting the PUFA contents. Further, the cpSNPs of these two genes showed higher explained PV than the cpSNPs of one gene on the contents of most PUFAs.

### 3.4. The Correlation between the PUFA Contents and the Expression Levels of Four Genes

In general, the expression levels of both *fads2a* and *fads2b* in the liver were significantly positively correlated with the contents of six same PUFAs (C20:3n-3, C20:4n-3, C22:5n-3, C20:4n-6, C22:4n-6, and C22:5n-6, FDR *p* values in Table 5), suggesting these two genes might cooperate to regulate the same PUFAs through expression.

However, we did not observe the significant correlation between the *elovl5a* expression and the PUFA contents. Similar scenario was observed between the *elovl5b* expression and the PUFA contents except C18:4n-3. These data indicated that the expression levels of these two genes might not influence the PUFA contents.

### 3.5. The Association of pSNPs on the fads2b Expression Levels

**Figure 2:**
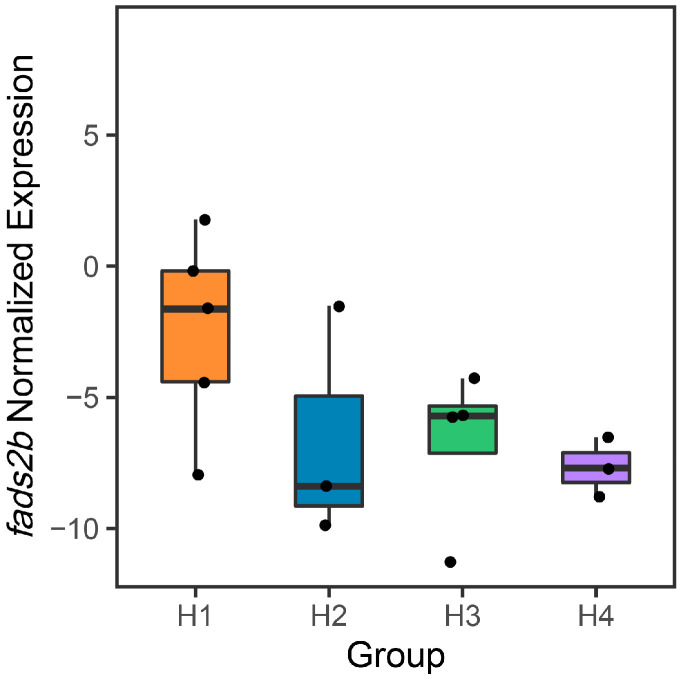
Whisker plots of the *fads2b* gene expression levels among different genotype combinations of four *fads2b* pSNPs (F2b.-104, F2b.-304, F2b.-700, and F2b.-785). H1: AT/CC/GG/AT. H2: AA/CT/GG/TT. H3: TT/CC/GG/AA. H4: TT/CC/GG/TT.

Because the *fads2b* expression levels were regulated by multiple transcriptional factors, we studied the correlation between the genotype combinations of four pSNPs (F2b.-104, F2b.-304, F2b.-700, and F2b.-785) and the *fads2b* expression levels. We observed 18 combinations where the individual numbers were over two (Supplementary Table S8). In the contrary, in the above expression study only four combinations (H1, H2, H3, and H4) corresponded to at least three individuals. For the first combination (H1) of AT/CC/GG/AT, the contents of seven PUFAs were higher than the ones in the samples of the other three combinations (Supplementary Table S9). Intriguingly, the *fads2b* expression levels of the H1 samples were also higher than the ones in the groups of H2, H3, and H4 (Figure 2). These data indicated that the genotypes of *fads2b* pSNPs were associated with both the PUFA contents and the *fads2b* expressions.

## 4. Discussion

*Fads2a*, *fads2b, elovl5a,* and *elovl5b* were reported to participate in the synthesis of PUFAs in common carp [38,39]. In this study, we scanned the pSNPs in these four genes in common carp and found higher genetic diversities of the promoter regions than the coding regions in these four genes.

Improving the desaturase activity of *fads2b* would be the top priority for selective breeding of common carp with higher PUFA contents. First, more pSNPs and cSNPs of *fads2b* were identified than in *elovl5b*. Second, the pSNPs of *fads2b* were associated with the contents of more PUFAs than *elovl5b.* Third, the explained PVs of *fads2b* cpSNPs was higher than the *elovl5b* cpSNPs.

We observed the joint effect of pSNPs and cSNPs in this study. Although the joint effect of cSNPs from two genes, *fads2b* and *elovl5b*, on the PUFA content were uncovered [25], none has studied the joint effects of pSNPs on the PUFA contents. We selected one SNP in one haplotype block to avoid overestimating the joint effect. The explained PV of each PUFA content was increased by combining the pSNPs and cSNPs, compared with one SNP. The coordination to improve the PUFA contents requires the simultaneous mutations in the promoters and coding regions in these two genes.

The associations of the pSNPs with the PUFA contents could be explained by their locations in the TFBSs. The TF of Sp1 could enhance the PUFA biosynthesis by increasing *fads2* and *elovl5* gene expression in rabbitfish (*Siganus canaliculatus*) [40]. Lack of the Sp1 binding site might decrease the promoter activity of *fads2* of carnivorous marine fish. The TF of CEBP (CCAAT/enhancer-binding protein) could enhance the *fads2* expression [41]. It could up-regulate the *fads2* promoter activity involving in the process of PUFAs biosynthesis in the large yellow croaker (*Larimichthys crocea*), and rainbow trout (*Oncorhynchus mykiss*) [42]. These pSNPs might alter the binding efficiency of TFs and then affect the target gene expression. In addition, the content of C20:3n-6 was associated with two pSNPs (F2b.-104 and F2b.-304). Because of the location of these two pSNPs in the TFBSs of Sp1 and HNF1, the co-association suggested the possible co-regulation of Sp1 and HNF1 in the biosynthesis of C20:3n-6. Similarly, two pSNPs (F2b.-104 and F2b.-785) were associated with the content of C22:5n-3, indicating the co-regulation of Sp1 and CEBP on this PUFA. More functional studies are required to further validate the effects of pSNPs.

The expressions of *fads2a* and *fads2b* were significantly positively correlated with the contents of multiple PUFAs (FDR *p* values listed in Table 5). We adopted two strategies to increase the reliability of correlation analysis, including increasing sample size and correcting the *p* value of each coefficient. Ching et al. suggested a sample size of 25 to achieve a power of 0.8 or greater [43]. Bottomly et al. collected 21 mice and compared gene expression between C57BL/6J and DBA/2J mouse strains [44]. In our study, we collected 44 samples to study the correlation between gene expression and the PUFA contents. It is reasonable that the higher expression level of targeted gene could generate more enzymes participating in the PUFA biosynthesis. Recent research showed the *fads2* expression influenced the PUFAs in breast milk [45], gilthead seabream [46], and pig [18]. The influences of pSNPs on the TFBSs might result in the change of both target expression and the PUFA contents. The H1 genotype combination of *fads2b* corresponded to higher *fads2b* expression, possibly generating more *fads2b* enzyme. The higher PUFA contents in the H1 samples might be due to more *fads2b* enzyme.

Herein, we investigated the polymorphisms in the promoters of *fads2a*, *fads2b*, *elovl5a*, and *elovl5b*. Together with our previous study, our data of explained PVs suggested that *fads2b* might be the major effect gene on the contents of multiple PUFAs and should be target for selective breeding to improve the PUFA contents. The increasing explained PVs with the pSNPs and cSNPs demonstrated that both changing the expressions of these genes in liver and altering their enzyme activities would influence the PUFA contents. We focused on the polymorphisms of these four genes. In the future, the genome wide association study would be used to identify more SNPs and indels related to the PUFA contents at the entire genome.

## 5. Conclusions

We identified the polymorphisms in the promoters of common carp *fads2a*, *fads2b*, *elovl5a*, and *elovl5b*. The association study identified six pSNPs in the latter three genes significantly related to the PUFA contents. The joint effects of pSNPs and cSNPs in either *fads2b* or *elovl5b* improved the explained percentages of PVs of the PUFA contents. Further, the joint effects of both genes had higher explained PVs than single gene. The pSNPs were in the predicted TFBSs. The expression levels of *fads2a* and *fads2b* were positively correlated with the contents of multiple PUFAs. The samples with one genotype combination corresponding to higher *fads2b* expression had higher PUFA contents. These pSNPs and cSNPs would be used to selectively breed common carp of higher PUFA contents.

## Figures and Tables

**Table 1:** Genetic diversities of the promoters of *elovl5a*, *elovl5b*, *fads2a*, and *fads2b*.

SNP	Ref/Alt	Ho	He	PIC	MAF	Genotype	Fre. Geno./No.
E5a.-122	A/G	0.115	0.216	0.193	0.123	AA	0.0661/15
						AG	0.1145/26
						GG	0.8194/186
E5a.-257	C/T	0.211	0.229	0.203	0.132	CC	0.7621/173
						CT	0.2115/48
						TT	0.0264/6
E5a.-264	A/T	0.458	0.436	0.341	0.322	AA	0.4493/102
						AT	0.4581/104
						TT	0.0925/21
E5a.-351	C/T	0.401	0.5	0.375	0.487	CC	0.3128/71
						CT	0.4009/91
						TT	0.2863/65
E5a.-430	G/T	0.075	0.088	0.084	0.046	GG	0.0088/2
						GT	0.0749/17
						TT	0.9163/208
E5b.-330	A/T	0.208	0.186	0.169	0.104	AA	0.7921/160
						AT	0.2079/42
E5b.-521	G/T	0.075	0.072	0.069	0.037	GG	0.9254/186
						GT	0.0746/15
F2a.-199	C/G	0.406	0.365	0.299	0.241	CC	0.0377/8
						CG	0.4057/86
						GG	0.5566/118
F2b.-99	G/T	0.066	0.119	0.112	0.064	GG	0.9035/206
						GT	0.0658/15
						TT	0.0307/7
F2b.-104	A/T	0.339	0.492	0.371	0.438	AA	0.2687/61
						AT	0.3392/77
						TT	0.3921/89
F2b.-304	C/T	0.246	0.306	0.259	0.189	CC	0.6886/157
						CT	0.2456/56
						TT	0.0658/15
F2b.-323	A/C	0.145	0.149	0.138	0.081	AA	0.0088/2
						AC	0.1447/33
						CC	0.8465/193
F2b.-609	A/G	0.085	0.174	0.159	0.096	AA	0.0538/12
						AG	0.0852/19
						GG	0.861/192
F2b.-650	A/C	0.049	0.114	0.107	0.061	AA	0.9148/204
						AC	0.0493/11
						CC	0.0359/8
F2b.-700	G/T	0.126	0.178	0.162	0.099	GG	0.8386/187
						GT	0.1256/28
						TT	0.0359/8
F2b.-721	G/T	0.143	0.171	0.156	0.094	GG	0.8341/186
						GT	0.1435/32
						TT	0.0224/5
F2b.-744	G/T	0.139	0.174	0.159	0.096	GG	0.8341/186
						GT	0.139/31
						TT	0.0269/6
F2b.-785	A/T	0.363	0.496	0.373	0.455	AA	0.3632/81
						AT	0.3632/81
						TT	0.2735/61
F2b.-880	C/T	0.137	0.41	0.326	0.288	CC	0.2192/48
						CT	0.137/30
						TT	0.6438/141

Ref: reference base. Alt: alternative base. Ho: observed heterozygosity. He: expected heterozygosity. PIC: polymorphism information content. MAF: minor allele frequency. Fre. Geno: frequency of genotype. No: sample number having this genotype.

**Table 2:** Association analysis of PUFAs contents using combining analysis of GLM and ANOVA in common carp.

Trait	SNP	GLM (Permutation)		ANOVA			
PUFA	ID	*p* Value	Marker PVE (%)	Genotype			*p* Value (FDR)
				MM	Mm	mm	
C18:3n-6	E5a.-257	6.12 × 10^−5^	8.55%	4.51 (CC)	4.53 (CT)	11.63 (TT)	1.16 × 10^−3^
C20:4n-3	E5b.-330	2.48 × 10^−3^	4.53%	0.32 (AA)	0.25 (AT)	--	4.7 × 10^−2^
C20:3n-6	F2b.-104	1.75 × 10^−3^	5.66%	3.4 (TT)	2.79 (AT)	2.46 (AA)	3.14 × 10^−2^
C22:5n-3	F2b.-104	5.16 × 10^−3^	4.74%	1.94 (TT)	1.72 (AT)	1.44 (AA)	9.29 × 10^−2^
C18:3n-3	F2b.-304	1.26 × 10^−2^	3.92%	1.04 (CC)	0.61 (CT)	0.94 (TT)	5.1 × 10^−2^
C20:3n-6	F2b.-304	2.92 × 10^−4^	7.16%	3.21 (CC)	2.5 (CT)	1.77 (TT)	5.54 × 10^−3^
C18:2n-6	F2b.-700	2.3 × 10^−3^	5.52%	23.95 (GG)	30.72 (GT)	27.78 (TT)	4.37 × 10^−2^
C20:3n-3	F2b.-785	3.75 × 10^−4^	7.11%	0.3 (AA)	0.29 (AT)	0.21 (TT)	7.12 × 10^−3^
C22:4n-6	F2b.-785	1.89 × 10^−3^	5.80%	0.76 (AA)	0.76 (AT)	0.56 (TT)	3.59 × 10^−2^
C22:5n-3	F2b.-785	4.85 × 10^−3^	4.88%	1.88 (AA)	1.84 (AT)	1.4 (TT)	9.22 × 10^−2^

Marker PVE: the explained percentage of PVs by markers. M: major allele. m: minor allele. The content of each PUFA was displayed as the mean value. The genotype was displayed in the bracket.

**Table 3:** The explained PV of cpSNP set on the contents of n-3 PUFAs.

cpsnp Set	C18:3n-3	C18:4n-3	C20:3n-3	C20:4n-3	C20:5n-3	C22:5n-3
E5b.-330	NA	NA	NA	4.53%	NA	NA
E5b.172	6.9%	NA	3.96%	7.12%	NA	3.7%
E5b.782	NA	NA	2.15%	NA	NA	NA
E5b.cpSNPs	11.31%	1.08%	11.84%	13.71%	4.43%	9.17%
F2b.-104	NA	NA	NA	NA	NA	4.74%
F2b.-304	3.92%	NA	NA	NA	NA	NA
F2b.-785	NA	NA	7.11%	NA	NA	4.88%
F2b.751	5%	NA	13%	11%	NA	11%
F2b.1197	NA	NA	4.3%	5%	NA	5%
F2b.cpSNPs	21.41%	8.69%	20.64%	17.91%	12.11%	14.42%
E5b.cpSNPs & F2b.cpSNPs	18.12%	15.74%	31.70%	20.64%	20.41%	21.79%

Note: E5b.cpSNP set contained one pSNP (E5b.-330) and two cSNPs (E5b.172 and E5b.782). F2b.cpSNP set included three pSNPs (F2b.-104, F2b.-304, and F2b.-785) and two cSNPs (F2b.751 and F2b.1197). E5b.cpSNPs & F2b.cpSNPs, harbored the mentioned pSNPs in E5b.cpSNPs and F2b.cpSNPs. NA meant that this SNP was not associated with the PUFA content.

**Table 4:** The explained PVE of cpSNP set on the content of n-6 PUFAs.

cpSNP Set	C18:2n-6	C18:3n-6	C20:3n-6	C20:4n-6	C22:4n-6	C22:5n-6
E5b.-330	NA	NA	NA	NA	NA	NA
E5b.172	2.86%	NA	3.37%	2.1%	3.44%	4.07%
E5b.782	NA	NA	4.48%	NA	1.83%	1.83%
E5b.cpSNPs	13.22%	1.29%	13.37%	3.45%	9.56%	11.65%
F2b.-104	NA	NA	5.66%	NA	NA	NA
F2b.-304	NA	NA	7.16%	NA	NA	NA
F2b.-785	NA	NA	NA	NA	5.80%	NA
F2b.751	NA	NA	11%	NA	7%	9%
F2b.1197	4%	NA	4%	NA	NA	3%
F2b.cpSNPs	10.08%	9.96%	16.99%	8.25%	12.63%	14.15%
E5b.cpSNPs & F2b.cpSNPs	12.37%	9.93%	28.65%	30.87%	43.74%	35.35%

**Table 5:** The correlation between the expressions of four genes in liver and the contents of 12 PUFAs.

PUFAs (%)	*fads2a*		*fads2b*		*elovl5a*		*elovl5b*	
	r	FDR *p* Value	R	FDR *p* Value	r	FDR *p* Value	r	FDR *p* Value
C18:3n-3	−0.091	0.558	−0.105	0.498	0.043	0.784	0.285	0.061
C18:4n-3	0.113	0.465	0.350 *	0.02	0.25	0.102	0.370 *	0.013
C20:3n-3	0.410 *	0.006	0.421 *	0.004	0.169	0.273	0.023	0.88
C20:4n-3	0.377 *	0.012	0.380 *	0.011	0.228	0.137	0.189	0.22
C20:5n-3	0.410 *	0.006	0.186	0.227	0.087	0.574	−0.058	0.708
C22:5n-3	0.456 **	0.002	0.362 *	0.016	0.063	0.682	−0.087	0.573
C18:2n-6	0.019	0.9	0.119	0.443	−0.044	0.778	0.208	0.175
C18:3n-6	0.151	0.329	0.028	0.858	−0.317	0.036 *	−0.349	0.02
C20:3n-6	0.144	0.352	0.244	0.111	0.099	0.521	0.21	0.171
C20:4n-6	0.354 *	0.018	0.434 *	0.003	0.201	0.191	−0.015	0.924
C22:4n-6	0.367 *	0.014	0.481 **	0.001	0.181	0.24	0.068	0.661
C22:5n-6	0.352 *	0.019	0.458 **	0.002	0.149	0.334	0.121	0.435

Note: r is Pearson’s correlation, and * represented the FDR adjusted *p* value < 0.05, ** represented the FDR adjusted *p* value < 0.01.

## Data Availability

The data presented in this study are available in Supplementary Materials.

## Supplementary Materials

The following are available online at https://www.mdpi.com/article/10.3390/biology12040524/s1, Table S1: The primers used for amplification of the promoter regions of four genes. Table S2: The primers used for qRT-PCR of four genes. Table S3: The pSNP genotypes of four genes in all individuals. Table S4: Predicted TFBSs around six pSNPs associated with the PUFA contents. Table S5: Association analysis of PUFAs contents with GLM-PCA. Table S6: ANOVA without the strain information and ANCOVA with the strain information. Table S7: Three haplotype blocks in *fads2b*. Table S8: The observed pSNP genotype combinations of *fads2b* and corresponding PUFA contents. Table S9: The expression levels of *fads2b* and the PUFA contents in different genotype combinations.
